# Effect of Annealing on the Mechanical Properties of Composites of PLA Mixed with Mg and with HA

**DOI:** 10.3390/polym17091207

**Published:** 2025-04-28

**Authors:** Carmen Sánchez González, Aurora Pérez Jiménez, Mauro Malvé, Cristina Díaz Jiménez

**Affiliations:** 1 Asociación de la Industria Navarra (AIN), Carretera Pamplona 1, E-31191 Cordovilla, Spain; aperez@ain.es (A.P.J.); cdiaz@ain.es (C.D.J.); 2 Department of Engineering, Public University of Navarra (UPNA), Av. Cataluña, s/n, E-31006 Pamplona, Spain; mauro.malve@unavarra.es; 3 Research Networking in Bioengineering, Biomaterials & Nanomedicine (CIBER-BBN), Av. Monforte de Lemos, 3-5, Pabellόn 11, Planta 0, E-28029 Madrid, Spain; 4 Instituto de Materiales Avanzados y Matemáticas (INAMAT2), Public University of Navarre (UPNA), Edificio Jerόnimo de Ayanz, Campus de Arrosadía, E-31006 Pamplona, Spain

**Keywords:** PLA, Hydroxyapatite, magnesium, annealing, reinforced materials, mechanical properties, additive manufacturing

## Abstract

Polylactic acid (PLA) is a bioresorbable and biocompatible material and is a promising alternative to the current materials used for permanent implants as it has osteosynthesis properties. However, this material has some drawbacks due to its low mechanical and thermal resistance after 3D printing. Extensive research has been conducted to improve the properties of this material, for example, with the addition of other compounds, such as magnesium (Mg) or Hydroxyapatite (HA). These reinforced materials have been shown to reduce the internal stress of the matrix of PLA, improving the thermal, optical and structural properties of the material, even though the performance achieved is lower than needed to be implanted. In addition, although it is known that the addition of Mg or HA affects the mechanical performance of the material, mechanical properties have not been studied in the literature. Thus, the aim of this study is to research the effect of thermal post-processing based on annealing of composites made of PLA with Mg and PLA with HA, manufactured by fused filament fabrication, with the goal of finding an improvement in the mechanical properties of these materials. As a result, different designs of annealing processes have been studied with different reinforced materials and their mechanical properties have been compared, studying axial traction and compression, radial compression as well as flexibility, among others. The comparative results achieved show the relevance of the design of the annealing process for the improvement of the mechanical properties of these materials.

## 1. Introduction

Additive manufacturing (AM) has emerged as a promising technique in industry, manufacturing customised 3D parts in a cost-effective manner. In this context, fused deposition modelling (FDM) is an economical and flexible process that consists of forming parts by extrusion of melted filament deposited layer by layer, to create the designed geometry without wasting material as in other technologies like moulding or casting [1]. In the biomedical field, FDM has been shown to be a potential alternative for manufacturing medical devices as it can print 3D unique fully adapted to the patient products, even with complex geometries, such as synthetic bone models [2,3], dentistry products [4] and tissue scaffolds [5]. Moreover, FDM allows for the use of a wide variety of biocompatible materials compared to other AM techniques, which makes it possible to choose the material with the most optimal properties for the desired application, as long as the melting point and viscosity are low enough to enable extrusion [1,6], reducing the FDM market mainly to thermoplastics. At a time when the environment is an important issue, this technology also allows for the use of sustainable and environmental materials, including biodegradable and bio-based materials. In this field, polylactic acid (PLA) stands out among all the FDM materials as one of the most promising and used biodegradable polymers [4,6,7,8,9,10].

PLA is a bioabsorbable and biocompatible thermoplastic made of natural resources such as corn or sugar cane [11,12]. Among its features, it is recognised as a safe material for clinical use [3], approved by the U.S. Food and Drug Administration [13], which highlights its thermal stability, elasticity and non-toxicity [12]. However, this semi-crystalline polymer presents some drawbacks, such as high hydrophobicity, poor bioactivity, low degradation rate and, although the mechanical properties are favourable with an elastic module similar to bone, they are not sufficient for functionality [6,12,13,14,15]. In addition, taking into account the weak bonds created during the material deposition [7], most of the 3D printed parts are used only for prototypes and not as a final part [1,16]. Due to the optimal relation between the ease of printing PLA and the quality of the parts, even for complex geometries, there are multiple manufacturers of filament spools and printers for this widely used material, which leads to the fact that there is no well-established method to extrude PLA [8]. Therefore, different authors agree that controlling the composition, sintering process, crystallinity and molecular weight of PLA is key to optimising its properties, demonstrating that small variations have a direct impact on the main properties of this widespread polymer, in particular mechanical strength, degradation rate and bioactivity [10,17,18,19]. Among the different alternatives for finding the improvement, adjusting the formula of PLA composites seems to be a method to find a biomechanical improvement [18], therefore, different authors have tested different combinations, such as PLA with Zirconium Dioxide (ZrO_2_) [4], poly(ε-caprolactone) (PCL) [11] and poly(3-hydroxybutyrate) (PHB) [8]. Two of the most promising combinations are PLA–Hydroxyapatite [18] and PLA–Magnesium [13,15]. These two materials are particularly interesting for bone regeneration applications, as they are resorbable by the human body, promote bone regeneration and can be 3D printed using FDM methods, allowing professionals to obtain cost-effective and customised parts for technical applications such as orthopaedic implants [3,15].

Hydroxyapatite (HA) is a biocompatible ceramic obtained from natural resources. Its surface simulates the appearance of bones and teeth [6], is highly bioactive and promotes the adhesion of proteins [6]. Nevertheless, it exhibits poor mechanical strength, brittleness and a slow degradation rate [12,14]. However, when mixed in a PLA matrix, it might create a biocompatible and resorbable composite and stands out as one of the most exciting combinations in the medical field, as it enhances osteoconduction and bone formation [2,3,14]. Regarding internal geometry, the introduction of HA reduces the accumulation of defects of PLA [2], without generating internal porosity [12]. In terms of functionality, it increases bioactivity and osteoinductivity, presenting good absorbability, hydrophilicity and biocompatibility [1,3,14]. Finally, regarding mechanical properties, it increases the crack resistance, compressive strength, bending strength and elastic modulus [12,18,20]. Despite the improvements of this material, the optimum properties are not yet achieved [14]. In particular, it is estimated that for simulating the human cortical bone, the HA wt. should be higher [2,3,12], a complicated parameter as it leads to an increase in porosity that deteriorates mechanical resistance [12] and printing is more challenging when the wt.% of HA is increased [2,3].

On the other hand, magnesium (Mg) is a biodegradable and biocompatible metal that is essential in the human body, as it plays a key role in the metabolism [15]. Although Mg shows a high mechanical strength and stiffness, with an antibacterial property to prevent infections [21], it is limited by its high degradation rate. Accordingly, some authors have found that the mixture of PLA with Mg is favourable; Mg neutralises the hydrolysis of the PLA matrix while PLA stabilises Mg and controls its degradation rate and corrosion, thus demonstrating the combination of Mg-reinforced PLA (PLA–Mg) composite as a powerful alternative [13,21,22,23]. In terms of mechanical properties, the composite improves compressive strength and stiffness [15]. Regarding functionality, PLA–Mg improves osteoconduction and osteointegration [23]. However, although Mg could be used as a multifunctional filler, increasing mechanical, degradation and biological properties [24], this material still has certain disadvantages, because the presence of Mg still produces cracks in the structure, releasing oxygen that can cause irritation and the loss of 60% of mechanical strength in only 28 days [21].

Although the composites introduced improve the performance of final pieces in comparison to raw PLA, a feasible material has not been found yet. As a result, post-processing seems to be required for improving the properties [8], thermal annealing being a simple and highly efficient method [17]. Annealing consists of heating the polymer at a temperature higher than the glass transition (Tg) and lower than the melting point (Tm) for a specific time [16] to boost diffusion between particles, which leads to molecular reorganisation, decreasing imperfections between layers [16] and increasing the degree of crystallinity [3,8,17], which is directly related to mechanical properties because of the semicrystalline structure of these materials [25,26]. As a result, it reduces the residual stress caused during the manufacturing process [7], leading to modification of the chemical and physical properties, including optical, thermal, electrical, mechanical and rheological performance, as well as hydrolysis and degradation rate [7,17,27].

Some authors highlight the benefits of composites of PLA–Mg and PLA–HA over pure material [3,12,13,14,15,18,20,21], while others focus on optimising the manufacturing process or the post-processing of composites [8,16]. However, although in the literature it is mentioned that composites with concretions higher than 5% should be improved, as well as the FDM process optimised [17], not many articles that combine both techniques have been found at the time of writing, and other authors agree that these materials are not yet thoroughly studied [3,22]. The crystallisation process of polylactic acid chains is a widely studied topic, since different methods, among which the controlled cooling rate after annealing and the introduction of nanoparticles stand out, have been used to increase the degree of crystallinity of these monomer units. In particular, changes in composition caused by these treatments, as well as the improvement of physical and chemical properties, are well-known and documented, but the reference to the mechanical properties that will directly determine which material can be used for each application and working conditions is often lost [5,7,9]. Therefore, the aim of this study is to evaluate the effects of annealing on the mechanical properties of the composites made of PLA mixed with HA and Mg, both with concentrations higher than 5%, to find an improvement in their mechanical performance to optimise the use of these composites in medical applications.

## 2. Material and Methods

**Figure 1:**
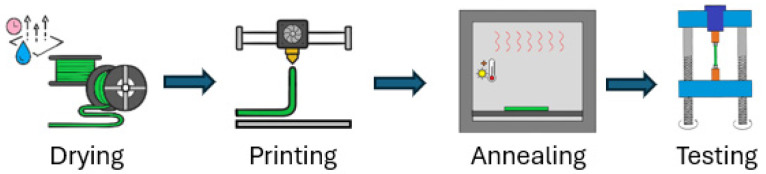
Experimental process.

This experimental set-up consists of drying the material prior to 3D printing it, to thermally post-process it to compare their mechanical properties (Figure 1).

### 2.1. Material

Three commercial PLA composite materials (Colfeed4print SL, Madrid, Spain) are used in this research. The materials are sold in 1.75 mm diameter filament spools. Technical specifications of these composites are detailed in Table 1. The first material, hereinafter PLA–HA20, is a composite based on PLA and HA (40 wt.%/20 vol.%). The second material, PLA–HA50, is a mix of PLA and HA (72 wt.%/50 vol.%). Finally, the third material, PLA–Mg10, is composed of PLA and Mg (20 wt.%/15 vol.%).

### 2.2. Specimen Printing

**Figure 2:**
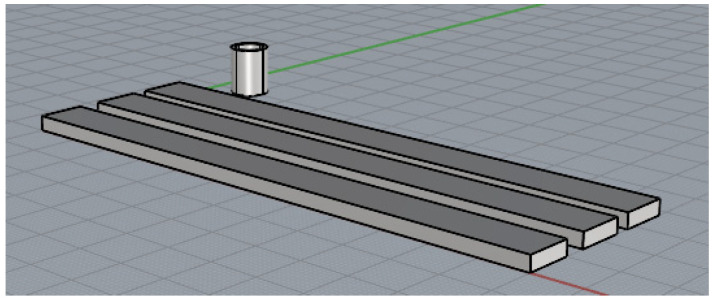
Design of test specimen.

**Figure 3:**
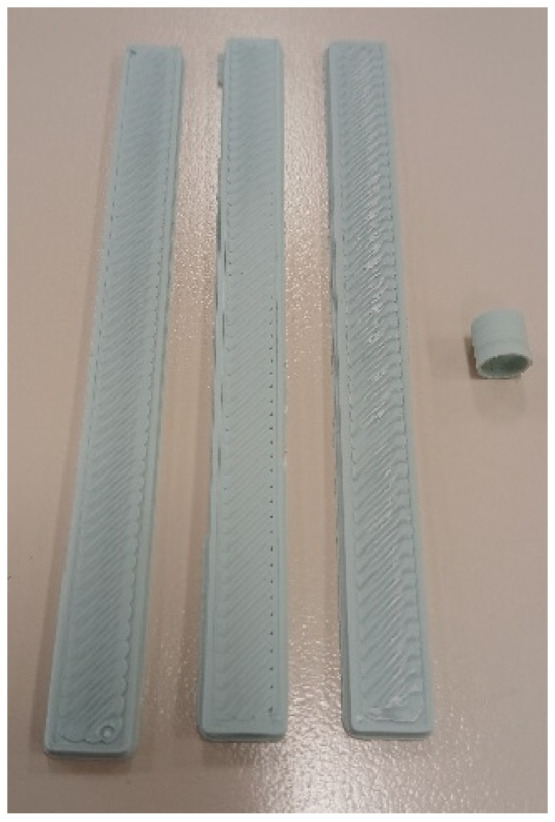
Samples of PLA–HA50 rectangular and tubular specimens to be tested.

For each material, it is proposed to manufacture three rectangular specimens and one tubular specimen. The specimens were not designed according to the ISO standards, because some authors and our own experience indicate that the section changes in FDM-printed specimens with thermoplastics are fragile points that directly influence mechanical tests [28]. For this reason, specimens are designed without changes of section, with dimensions of 120 mm long, 10 mm wide and 3 mm deep for axial tensile, compressive and flexure tests, and with dimensions of internal radius of 8 mm and external radius of 6.5 mm, with a length of 8 mm for the compressive radial test (Figure 2). A commercial computer-aided design (CAD) software is used for the 3D design (Autodesk Inventor Professional 2025-Español, Autodesk Inc., San Francisco, CA, USA) that is then converted into stereolithographic (STL) format. In addition, another commercial computer-aided manufacturing (CAM) software is used to set the printing parameters (Simplify3D^®^ version 5.1.2, Cincinnati, OH, USA). An attempt has been made to use the same printing conditions for all materials, as these parameters have a strong influence on the values of mechanical properties [29]. The printing parameters are included in Table 2. 3-Dimensional printer NX PRO Dual Filament–Filament (TUMaker, Indart 3D, Irún, Spain) is used for manufacturing the specimens to conduct the proposed test (Figure 3). Prior to melting the extrusion, the materials are dried for 2 h at 50 °C in a spool drier (eBox Lite, eSun, Shenzhen, China).

### 2.3. Post-Processing Treatment

**Figure 4:**
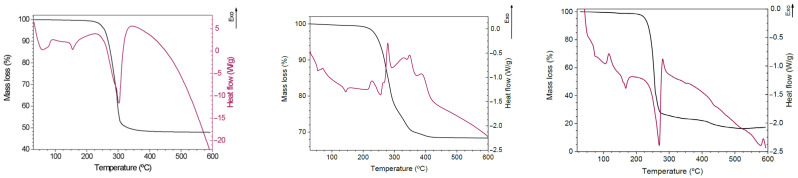
Thermal behaviour of, from left to right, PLAHA20, PLAHA50 and PLAMg10. Source: Colfeed SL.

**Figure 5:**
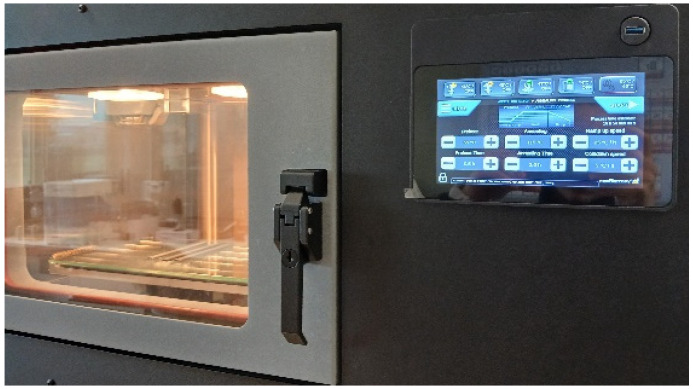
Annealing of PLA–Mg specimens in the Minifactory Ultra 2 printer.

**Figure 6:**
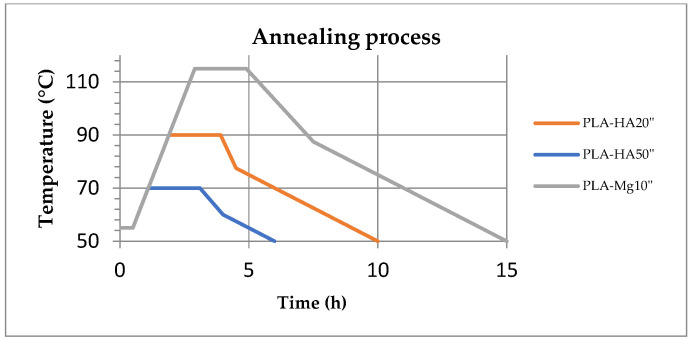
Annealing conditions for PLA–HA20″, PLA–HA50″ and PLA–Mg10″.

With respect to the post-processing of the specimens, this study evaluates three different conditions, which are called process 1, process 2 and process 3. Process 1 refers to the unprocessed sample. Process 2 refers to the sample annealed at a constant temperature, and cooled directly to room temperature. Finally, process 3 consists of maintained preheating to stabilise the sample for a given time, which is then heated progressively to the annealing temperature and, once annealed for the set time, a controlled cooling to room temperature is performed. For each post-processing condition, one specimen of each type and of each material is analysed. Some authors have found that prolonging the time or raising the annealing temperature does not have any effect on the matrix [30,31]. Moreover, considering that less than 60 min is needed to achieve 50% of the final crystallinity [17], the samples are subject to annealing for 2 h. Temperatures are chosen according to the thermal behaviour of each material provided by the manufacturer (Figure 4), and corroborated by studies carried out by other researchers [16,29,31]. Annealing is performed between the glass transition and melting temperature of each material [16]. First, the samples are analysed without any additional post-treatment steps by storing the samples at room temperature (23 °C–50% humidity) after printing (hereinafter referred to as PLA–HA20, PLA–HA50 and PLA–Mg10). Second, samples are subjected to constant annealing for 2 h with a temperature of 90 °C for PLA–HA, 70 °C for PLA–HA50 and 115 °C for PLA–Mg10 (hereon PLA–HA’, PLA–HA50′ and PLA–Mg10′, respectively), using the chamber of the Minifactory Ultra 2 (miniFactory Oy Ltd., Seinäjoki, Finland) as a conventional oven, and cooling to room temperature. Finally, the controlled annealing system of the Ultra 2 3D FDM printer (miniFactory Oy Ltd., Finland) (Figure 5) was used. For this process, it is worth mentioning that the material is, first of all, preheated for 30 min at 55 °C, then the temperature is raised up to the annealing temperature at a speed of 25 °C/h, to be annealed at the same conditions of 90 °C, 70 °C and 115 °C for 2 h for each of the above-mentioned materials (samples are called PLA–HA20″, PLA–HA50″ and PLA–Mg10″) and finally slowly cooled to room temperature at a rate of 5 °C/h. The annealing curves are shown in Figure 6. All specimens were stored under environmental conditions until they were mechanically tested.

### 2.4. Determination of Mechanical Properties

**Figure 7:**
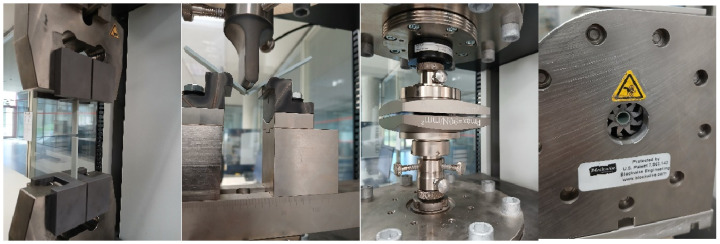
Tensile, flexure, axial compression and radial compression test, from left to right.

Tensile, compression, flexural and radial strength tests were conducted using a Zwick Roell universal testing machine (Zwick GmbH & Co. KG, Ulm, Germany) (Figure 7). The axial testing conditions are based on ISO standards, detailed in Table 3, for plastic materials, whereas the radial testing conditions are completed under a cyclic testing program of Zwick Roell based on ASTM F3067 [32], to measure radial compression force on stents. The tensile test consists of clamping the two ends of the specimen at a distance of 10 mm on each side and straining the material lengthwise until it breaks. Initially, clamps are 18.02 mm apart and a 0.1 MPa preload is applied. The test speed is 50 mm/min and the tensile modulus speed is 1 mm/min. Second, a three-point flexure test is performed, which consists of placing the ends of the specimen on two supports and applying force at the midpoint until it is deformed or broken. The flexure modulus speed is 1 mm/min and the test speed is 5 mm/min, applying a preload of 0.1 MPa. The third test, the compressive axial test, consists of compressing the specimen between two plates to study its response. As in the previous test, a preload of 0.01 MPa with a compression module speed of 1 mm/min is used, 10 mm/min being the test speed. Finally, the radial compression test consists of squeezing the specimen radially, until its external radius is reduced by 2.5 mm at a test speed of 10 mm/min and its forces are studied.

## 3. Results and Discussions

All materials used are bioabsorbable in the human body. In this article, the mechanical properties of the specimens are studied before use, so the working conditions are not taken into account, and it is assumed that the materials have not started to degrade.

To begin with, opposing results can be found regarding material properties in the literature, both in composite and post-processing treatments. Some authors mention that, given the large number of parameters to be taken into account for material mixing and composition, extrusion and post-processing, mechanical results can be opposite even though the concept is the same, due to small variations in these processes [30,31]. The results shown in this section are calculated as the average of three samples tested for each material, in the three different post-processing study cases.

### 3.1. Structure and Morphology of Samples

**Figure 8:**
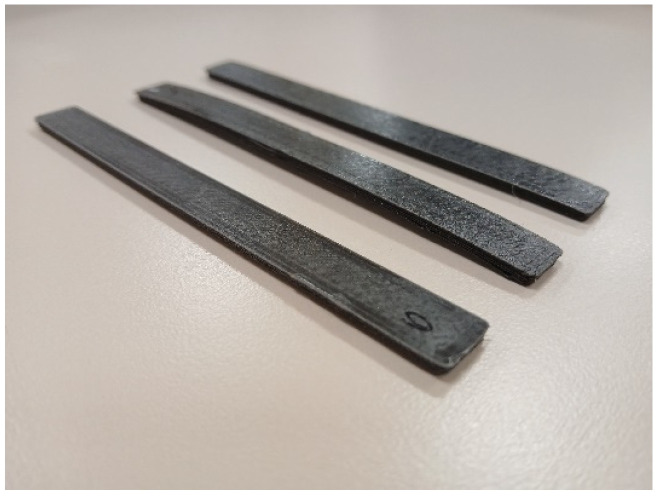
Sample curvature after post-processing. From left to right: PLA–Mg 10, PLA–Mg 10′, PLA–Mg 10″.

All samples have an addition of particulates, ceramic or metal, higher than 5%. Visually, there is a difference in the colour of the samples depending on the concentration of HA. The 20% HA–PLA (PLA–HA20) composite has a greenish-blue colour, and the 50% HA composite (PLA–HA50) is lighter blue. With respect to the influence of post-processing, no differences in texture, colour, shape or dimension were observed in the HA composites, before or after annealing [7]. However, it has been observed that the Mg composite samples, despite maintaining the same dimensions-texture-colour, bend when subjected to heat treatment. The curvature is more pronounced in the case of abrupt cooling (PLA–Mg10′) and lighter in the case of controlled cooling (PLA–Mg10″) (Figure 8).

### 3.2. Mechanical Properties

#### 3.2.1. Tensile Test

**Figure 9:**
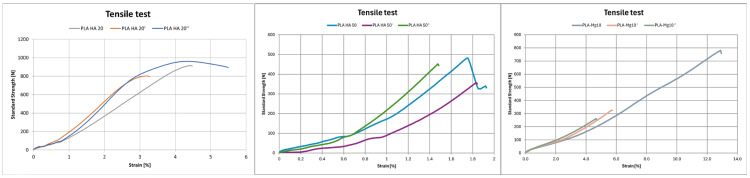
Tensile test results of (**a**) PLA HA20; (**b**) PLA HA50; (**c**) PLA Mg10.

All materials tested, show brittle behaviour (Figure 9), as they break at low deformation (less than 4.5%) without showing a yield point (Table 4). It is worth mentioning that the percentage of HA in the PLA matrix directly influences the tensile strength (s_m_) of the samples [12]. Although the longitudinal modulus of elasticity (E_T_) is similar for both composites (376.91 MPa for PLA–HA20 and 369.65 MPa for PLA–HA50), which means that they exhibit a comparable stiffness under the same tensile stress, less force (41.47% less) is required to break the material PLA–HA50 in comparison to PLA–HA20. The PLA–HA20 sample is three times more resistant to tensile stresses, demonstrating higher pre-breakage strengths and allowing higher strains at which strength is achieved (e_m_) than PLA–HA50, which shows a more brittle behaviour. With respect to the influence of annealing on these samples, rapid cooling worsens the tensile strength of the PLA–HA20″ and PLA–HA50″ composites (12.31% and 25.84%, respectively), embrittling their behaviour, while the samples with controlled annealing show similar performance in terms of strength and deformation (PLA–HA20″ is 1.44 MPa higher and PLA–HA50″ is 1.04 MPa lower than the reference), although E_T_ are lower in both cases. On the other hand, an increase in E_t_ of PLA–Mg10″ and PLA–Mg10′ with respect to the sample without post-processing PLA–Mg10 is observed, which leads to an increase in the stiffness of the post-processing materials. Nevertheless, annealed samples withstand less force before they break, breaking at 17.13% strength in the case of PLA–Mg10″ and at 35.26% strength in the case of PLA–Mg10′, with respect to the reference.

#### 3.2.2. Flexure Test

**Figure 10:**
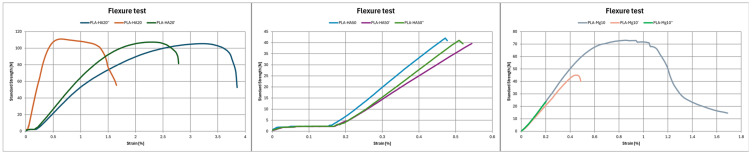
Flexure test results of (**a**) PLA HA20; (**b**) PLA HA50; (**c**) PLA Mg10.

As in the tensile test, PLA–magnesium composite loses stiffness when subjected to heat treatment (Figure 10, Table 5). The flexural modulus (E_f_) and flexural strength (s_fm_) of rapidly cooled Mg (PLA–Mg10′) are lower than those of controlled cooling (PLA–Mg10″), both lower than those of the unprocessed material (PLA–Mg10). Specifically, the decrease in s_fm_ of each composite is 65.27% and 64.04%, respectively. On the other hand, once again, the percentage of HA affects the material properties. PLA–HA50 (s_fm_ = 27.67 MPa) material is much more brittle than PLA–HA20 (s_fm_ = 65.14 MPa), showing lower breaking strength. Within the influence of post-processing, PLA–HA20 samples behave in a similar manner, although it is true that there is a slight deterioration in the flexural strength, with the unprocessed sample PLA–HA20 being the most resistant (s_fm_ = 65.14 MPa), followed by the controlled post-processed PLA–HA20″ (s_fm_ = 61.77 MPa) and, finally, the PLA–HA20′ (s_fm_ = 60.88 MPa) sample is the most fragile. The opposite result is shown for PLA–HA50, which shows an improvement in brittleness. The PLA–HA50″ matrix is 14.75% and the PLA–HA50′ is 10.19% more resistant to bending than PLA–HA50, which is the most brittle material.

#### 3.2.3. Compressive Test

**Figure 11:**
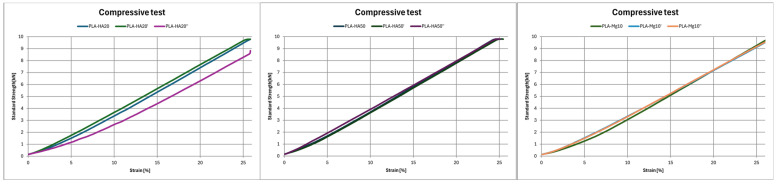
Compressive test results of (**a**) PLA HA20; (**b**) PLA HA50; (**c**) PLA Mg10.

Due to the limitations of the load cell (max. 10 kN) used and given the thermoplastic character and geometry of the materials used, in no case has the yield and/or break limit been reached (Table 6, Figure 11). As a result, the compressive modulus (E_c_) is defined as the slope of the straight line that fits the stress-strain curve in the linear region [2], whereas s_1_ is the compressive load at 1% of strain. In the case of composites formed by HA, in both cases it is observed that sudden annealing (PLA–HA20′ and PLA–HA50′) stiffens the material, increasing the E_c_ (16.2 MPa and 21.29 MPa, respectively), while controlled cooling (PLA–HA20″ and PLA–HA50″) softens the material (13.72 MPa and 16.37 MPa, respectively), so that for lower hardnesses there will be more deformation. On the other hand, for Mg composite, an increase in E_c_ after annealing is observed. E_c_ is 8.15% higher in the case of controlled annealing (PLA–Mg10″) versus sudden cooling (PLA–Mg10′), which is 8.16% higher than the specimen without post-processing. Therefore, the material PLA–Mg10 is stiffer after annealing, with an increase in compression strength [24].

#### 3.2.4. Radial Test

**Figure 12:**
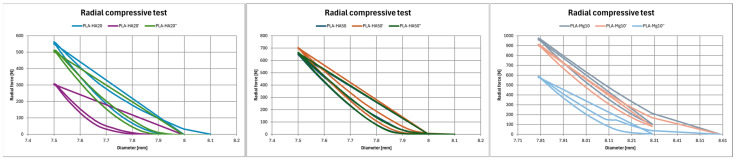
Radial test results (**a**) PLA HA20; (**b**) PLA HA50; (**c**) PLA Mg10.

The last parameter analysed is the radial compressive strength of tubular specimens (Table 7, Figure 12). In the case of HA, it is observed that controlled annealing increases the compressive radial strength in both cases. For PLA–HA20, the supported radial force increases five times for the controlled annealing specimen (PLA–HA20″) and decreases slightly (11.66%) in the case of sudden cooling (PLA–HA20′). On the other hand, with respect to the sample with a higher percentage of HA, the supported radial force is increased by 8% with respect to PLA–HA50′ and by 31.46% with respect to PLA–HA50″. However, in the case of magnesium, the opposite is observed. The annealing decreases the supported radial strength, being the controlled annealing PLA–Mg10″ 39.68% less resistant and the abrupt annealing PLA–Mg10′ 6.42% less resistance than the reference PLA–Mg10.

### 3.3. Annealing Influence

Although in the literature it appears that annealing improves all mechanical properties, including tensile modulus and strength, flexural strength and compressive modulus, among others [5,7,9,10,27,33], the mechanical results above show that there is not a perfect combination that improves all mechanical properties at the same annealing time and temperature. In this study, annealing improves mechanical properties in the case of HA and worsens them in the case of Mg, except for the compression test, where the opposite occurs. This statement could be considered consistent with a minority of authors [31] and could corroborate that the crystallisation of materials may not be the main cause of increased mechanical performance [3], which, in part, can be justified by the fact that very high concentrations of fillers are being used [17]. Moreover, it could be observed that changes in mechanical properties might be related to the temperature and cooling rate of the annealing process. Contradictorily, the existing literature considers these properties as static and focuses on the annealing time, not considering the cooling rate [7]. Regarding the HA composites, the mechanical test results of HA composites with controlled cooling at a rate of 5 °C/h are better than those with abrupt cooling (directly to room temperature after annealing), therefore, it appears that ramp-down cooling allows for better reorganisation of the material particles, which results in better mechanical properties. As for the Mg composite, the worsening of mechanical strength may be due to two factors: the high Mg content of the composite, since a content higher than 7.5% seems to worsen the mechanical properties, and the heat treatment. These two factors seem to agitate the particles, creating large defects and concentrating stresses, thus generating a weaker material [24].

## 4. Conclusions

This study focuses on the mechanical analysis of two composites with a high filler concentration of nanocomposites, PLA–HA and PLA–Mg, under different annealing conditions, which are of interest for medical applications in which temporary resorbable implants are required.

On the one hand, it can be determined that the composition of the PLA mixture has a stronger influence on the mechanical performance than the thermal treatment of the polymer, since it is observed that the wt.% of HA added has a direct effect on the performance, worsening the mechanical properties when exceeding the concentration of this ceramic. As a result, PLA with 50% HA presents worse mechanical properties than the 20% HA composite, being a much more fragile material even though the osteosynthesis may be considered better due to the higher concentration of hydroxyapatite. In terms of cooling rate and mechanical properties, in the flexure, compressive radial and tensile analyses of PLA–HA20, it has been observed that the mechanical behaviour is consistent with the physical and chemical behaviour described in the literature since controlled cooling gives better results than cooling at room temperature after annealing. As a conclusion, it seems advisable to define heating and cooling rates for each combination of polymers.

Thus, we have been able to verify that annealing is detrimental to the PLA and Mg composite, making it more fragile and worsening its tensile and flexural strength, as well as the radial force supported.

In conclusion, it can be determined that there is no gold standard combination of material composition and an annealing process that enhances all mechanical properties at the same time. Therefore, it will be necessary to define the desired properties and requirements for each application in order to determine the material with the most optimal composition and treatment, according to mechanical and functional needs.

## Figures and Tables

**Table 1:** The materials’ properties. Information provided by Colfeed SL, Madrid, Spain.

	PLA–HA20	PLA–HA50	PLA–Mg10
Density (g/cm^3^)	1.63	2.19	1.32
Linear density (g/cm)	0.039	0.053	0.031
Chemical formula	Ca_5_(PO_4_)_3_OH	Ca_5_(PO_4_)_3_OH	Mg
Ca/P ratio	1.74	1.74	NA
Purity (%)	99.5–99.8	99.5–99.8	>99.8%
Glass transition temperature (°C)	62	62	58
Melting temperature (°C)	156	156	150
Degradation temperature (°C)	320	320	320

**Table 2:** Printing properties. Information provided by Colfeed S.L, Madrid, Spain.

	PLA–HA10	PLA–HA50	PLA–Mg10
Printing temperature (°C)	160	160	160
Hot pad (°C)	45	45	45
Print speed (mm/s)	20	30	15
Layer height (mm)	0.4	0.4	0.4
Nozzle diameter (mm)	0.8	0.8	0.8
Infill pattern	Rectilinear	Rectilinear	Rectilinear
Infill angle (°)	45	45	45
Infill density (%)	100	100	100

**Table 3:** Testing Standards.

Normative	Test
ISO 527-1:2019	Plastics—Determination of tensile properties
ISO 178:2019	Plastics—Determination of flexural properties
ISO 604:2002	Plastics—Determination of compressive properties
ASTM F3067	Standard Guide for Radial Loading of Balloon-Expandable and Self-Expanding Vascular Stents

**Table 4:** Tensile test results.

	E_t_	s_m_	e_m_
	MPa	MPa	%
PLA HA 20	376.91	28.36	4.428
PLA HA 20′	324.37	24.87	3.175
PLA HA 20″	184.89	29.80	4.311
PLA HA 50	369.65	16.60	1.757
PLA HA 50′	77.60	12.31	1.825
PLA HA 50″	277.00	15.56	1.476
PLA–Mg 10	294.91	26.36	3.755
PLA–Mg 10′	335.01	9.29	1.854
PLA–Mg 10″	417.99	4.51	1.408

**Table 5:** Flexural test results.

	E_f_	s_fM_	e_fM_
	MPa	MPa	%
PLA–HA20	1227.27	65.14	2.56
PLA–HA20′	487.06	60.88	2.22
PLA–HA20″	549.26	61.77	3.42
PLA–HA50	2310.78	27.67	0.65
PLA–HA50′	3413.31	30.49	0.51
PLA–HA50″	2490.77	31.75	0.51
PLA–Mg10	450.06	53.79	3.13
PLA–Mg10′	346.55	18.68	0.98
PLA–Mg10″	429.36	19.34	0.99

**Table 6:** Compressive test results.

(MPa)	PLA–HA20	PLA–HA20′	PLA–HA20″	PLA–HA50	PLA–HA50′	PLA–HA50″	PLA–Mg10	PLA–Mg10′	PLA–Mg10″
E_c_	15.36	16.2	13.72	16.50	21.29	16.37	11.30	12.99	14.05
s_1_	0.283	0.302	0.228	0.291	0.354	0.295	0.235	0.253	0.272

**Table 7:** Radial compression test result.

	RF_max stand_
	N/mm
PLA–HA20	18.81
PLA–HA20′	16.80
PLA–HA20″	50.43
PLA–HA50	66.37
PLA–HA50′	71.50
PLA–HA50″	87.25
PLA–Mg10	96.97
PLA–Mg10′	90.75
PLA–Mg10″	58.59

## Data Availability

The original contributions presented in this study are included in the article. Further inquiries can be directed to the corresponding author.
